# Synthesis of Some New 3, 5-Bis (substituted) Pyrazoles and Isoxazoles Based on (*N’^1^E, N’^3^E)- N’^1^, N’^3^-Bis* (3, 4, 5-substitutedbenzlidene) Malonohydrazide under Solvothermal Conditions

**Published:** 2010-03

**Authors:** Ebraheem Abdu Musad, Kuriya Madavu Lokanatha Rai, Kullaiah Byrappa

**Affiliations:** 1 *Department of Chemistry, University of Mysore, Manasagangotri, Mysore, India;*; 2 *Department of Geology, University of Mysore, Manasagangotri, Mysore, India*

**Keywords:** solvothermal, pyrazoles, isoxazoles

## Abstract

The new 3,5-(substituted) pyrazoles and isoxazoles were prepared by reaction of *(N’^1^E, N’^3^E)- N’^1^, N’^3^-bis* (3,4,5-substitutedbenzylidene)malonohydrazide with hydrazine hydrate and hydroxylamine hydrochloride respectively under solvothermal conditions involving an ecofriendly method without any environmental pollution, the yield are in the range of 75–96%. The structure of the new compounds were established using elemental analysis, IR, ^1^H NMR, ^13^C NMR.

## INTRODUCTION

**Figure 1:**
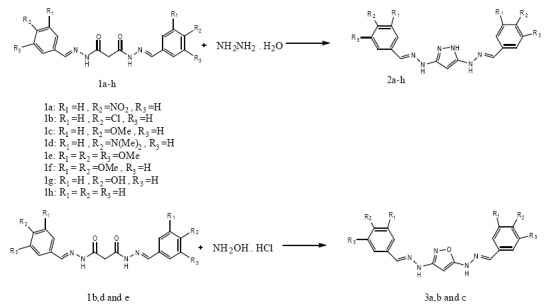


Synthesis of pyrazole and isoxazole derivatives has been a subject of consistent interest because of the wide applications of such heterocycles in pharmaceutical as well as agrochemical industry. Numerous compounds containing pyrazole and isoxazole moieties have been shown to exhibit antihyperglycemic, analgesic, anti-inflammatory, antipyretic antibacterial, antiviral, antitumor, antifungal and antidepressant activities (1–13). They are also useful intermediate for many industrial products (14, 15). A lot of synthetic methods for pyrazoles and isoxazoles have been developed. However these methods are usually carried out in organic solvents and many of them reactions require long reaction times, high temperatures, or inconvenient reaction condition for their execution and are often accompanied by tedious chromatographic techniques for purification of the desired products. Recently Rai *et al* have developed a simple method for the organic synthesis in the presence of a salen metal catalyst. The chemistry of solvothermal reactions involves two major factors namely, high temperature and pressure. “Solvothermal reactions can be defined as reaction in closed system, in the presence of solvent at a temperature higher than its boiling point. Consequently these reactions can be developed in supercritical or in sub critical conditions” (16). Some of the reactions, which proceed at slow rates under normal pressure, can be enhanced by increasing the pressure. This can be achieved by heating the reaction mixture under sealed condition. The development of solvothermal reaction is of interest because they offer the possibility of environmentally benign reaction conditions by reducing the burden of organic solvent disposal. Solvothermal process involves the heterogeneous chemical reaction, which occur at solid-liquid or solid-liquid-gas interfaces under high temperature and high pressure. Rai and Linganna successively used thiourea as thionating agent for (i) the conversion of 1,3,4-oxadiazole to1,3,4-thiadiazole under solvothermal condition (17) by heating in a sealed tube at 100°C. (ii) the conversion of esters to thioesters under solvothermal condition using More’s autoclave (18). Besides, Rai *et al* (19) successfully converted aldehyde semicarbazones to bishydrazones by thermolysis under pressure using ethanol as solvent in a sealed tube. These facts prompted us to apply this technology for the synthesis of 3,5-bis(substituted)pyrazoles and isoxazoles based on cyclocondensation of (*N’^1^E, N’^3^E)- N’^1^, N’^3^-bis* (3,4,5-substitutedbenzylidene)malonohydrazide with hydrazine hydrate and hydroxylamine hydrochloride via solvothermal mode (Figure 1).

## RESULTS AND DISCUSSION

**Figure 2:**
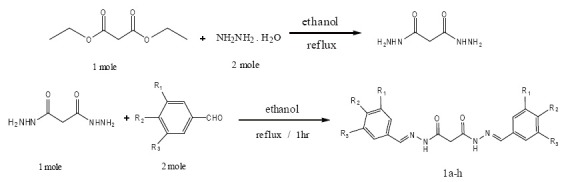


Synthesis of malonyldihydrazide was prepared by reacting the diethylmalonate (1mole) with hydrazine hydrate (2mole) (Figure 2). The desired starting materials (1a-h) were prepared in high yield by reacting ethanol solution of dihydrazide (1mole) with different benzaldehyde derivatives (2mole). The mixture was refluxed for 1hour and filtered, washed with ethanol and dried over desiccator (Figure 2).

Syntheses of pyrazoles (2a-h) were undertaken first. Thus, when (*N’^1^E, N’^3^E)- N’^1^, N’^3^-bis* (3,4,5-substitutedbenzylidene)malonohydrazides (1a-h) were reacted with hydrazine hydrate in the absence of solvent under solvothermal condition using More’s autoclave at 100°C for 1hour, Products (2a-h) were obtained in good to excellent yield. In the case of reaction between 1a and hydrazine hydrate the yield is relatively low, this is ascribed to lower reaction activity of compound 1a due to strong electron-withdrawing effect of the nitro groups. Similarly treatment of (*N’^1^E, N’^3^E)- N’^1^, N’^3^-bis* (3, 4, 5-substitutedbenzlidene) malonohydazides (1b,d and e) with hydroxylamine hydrochloride under solvothermal condition using More’s autoclave at 150°C for 3hours afforded compounds (3a-c) in good yield. The structures of isolated products were established with the help of spectral and analytical data.

## CONCLUSIONS

We report the reactions of (*N*’^1^ *E*, *N’^3^E*) - *N*’^1^, *N*’^3^-bis (3,4,5-substitutedbenzlidene) malononhydrazides with hydrazine hydrate and hydroxylamine hydrochloride under solvothermal conditions, a variety of pyrazole and isoxazole derivatives have been obtained in high yields.

### Experimental

All reagents were purchased from commercial sources. ^1^H NMR (300 MHz) and ^13^C NMR (100 MHz) spectra were recorded on a Bruker av300 spectrometer in CDCl_3_ or DMSO-d_6_ and TMS is used as internal reference. IR spectra (KBr) were recorded on a FTIR spectrophotometer in the range of 400–4000 cm^−1^. Elemental analyses were carried out on an Elementor vairo-EL instrument. Melting points are uncorrected. The starting materials (1a-h) were prepared according to the method cited in literature (20).

### Synthesis of 3, 5-bis(substituted) pyrazoles (2a-h) – General procedure

A mixture of (1a, 0.01mol) and excess of hydrazine hydrate were taken in autoclave reaction container (Teflon liner). The lid was placed and was lowered into the autoclave, the plates were kept over it and the autoclave was closed and tightened. It was kept in the oven at 100°C for 1hour. The products after cooling, were extracted into ethylacetate (20ml), washed thoroughly with water. The organic layer was dried over Na_2_SO_4_, solvent was evaporated and the product obtained as:

**2a: Solid 71% mp 98–100°C. Anal. Calc. for C_17_H_14_N_8_O_4_:** C, 51.78, H, 3.58, N, 28.42. **Found:** C, 51.46, H, 3.61, N, 28.08%.^1^H NMR (CDCl_3_); δ 4.10 (br, ^1^H, NH), 6.00 (s, 1H, C4), 7.70-8.30 (dd, 4H, Ar), 8.50 (s, 1H, -N=CH), 12.20 (br, 1H, NH). ^13^C NMR (100 MHz DMSO-d_6_): δ 77.80, 124.02, 125.20, 134.04, 143.40, 145.52, 151.56, 159.66. IR (KBr): υ(cm^1−^) 3426, 3291, 3073, 1600, 1512, 1336.

**2b: Solid 81% mp 168–171°C. Anal. Calc. for C_17_H_14_C_l2_N_6_:** C, 54.71, H, 3.78, N, 22.52. **Found:** C, 53.92, H, 3.63, N, 23.01%.^1^H NMR (CDCl_3_); δ 4.10 (br, 1H, NH), 7.30 (s, 1H, C4), 7.70-7.80 (dd, 4H, Ar), 8.70 (s, 1H, -N=CH), 11.80 (s, 1H, NH). ^13^C NMR (100 MHz DMSO-d_6_): δ 76.20, 123.58, 127.58, 131.34, 139.46, 146.79, 147.47. IR (KBr): υ(cm^1−^) 3113, 3050, 1589, 1486.

**2c: Solid 91% mp 138–141°C. Anal. Calc. for C_19_H_20_N_6_O_2_:** C, 62.62, H, 5.53, N, 23.06. **Found:** C, 62.44, H, 5.62, N, 22.80%.^1^H NMR (CDCl_3_); δ 3.80 (s, 3H, -OCH_3_), 4.20 (br, 1H, NH), 6.20 (s, 1H, C4), 6.90-7.70 (dd, 4H, Ar), 8.60 (s, 1H, -N=CH), 12.20 (br, 1H, NH). IR (KBr): υ(cm^1−^) 3369, 3166, 3077, 2950, 2841, 1581, 1422.

**2d: Solid 94% mp 145°C decomposes. Anal. Calc. for C_21_H_26_N_8_:** C, 64.59, H, 6.71, N, 28.70. **Found:** C, 64.42, H, 6.82, N, 28.50%.^1^H NMR (DMSO-d_6_); δ 2.99 (s, 6H, -N(CH_3_)_2_), 6.20 (s, 1H, C4), 6.70-7.40 (dd, 4H, Ar), 8.00 (s, 1H, -N=CH), 10.70 (s, 1H, NH). ^13^C NMR (100 MHz CDCl_3_): δ 40.19, 111.73, 123.24, 129.87 147.43, 152.10, 160.82. IR (KBr): υ(cm^1−^) 3192, 3134, 3077, 2911, 2811, 1615, 1527, 1437.

**2e: Solid 89% mp 114–117°C. Anal. Calc. for C_23_H_28_N_6_O_6_:** C, 57.02, H, 5.82, N, 17.35. **Found:** C, 56.91, H, 5.85, N, 16.82%.^1^H NMR (DMSO-d_6_); δ 3.80 (s, 9H, -OCH_3_), 6.60 (s, 1H, C4), 6.85 (s, 2H, Ar), 7.60 (s, 1H, -N=CH), 11.20 (s, 1H, NH). ^13^C NMR (100 MHz DMSO-d_6_): δ 55.90, 59.97, 78.20, 103.58, 128.24, 138.38, 144.42, 146.38, 153.40. IR (KBr): υ(cm^1−^) 3369, 3166, 3077, 2950, 2841, 1581, 1415, 1341.

**2f: Solid 87% mp 160–164°C. Anal. Calc. for C_21_H_24_N_6_O_4_:** C, 59.42, H, 5.70, N, 19.80. **Found:** C, 59.02, H, 5.85, N, 19.52%.^1^H NMR (CDCl_3_); δ 3.80 (s, 6H, -OCH_3_), 4.30 (br, 1H, NH) 6.20 (s, 1H, C4), 6.80-7.30 (dd, 2H, Ar), 7.60 (s, 1H, Ar) 8.60 (s, 1H, -N=CH), 12.20 (br, 1H, NH). IR (KBr): υ(cm^1−^) 3177, 3073, 2935, 1595, 1457, 1266.

**2g: Solid 83% mp 118–121°C. Anal. Calc. for C_17_H_16_N_6_O_2_:** C, 60.71, H, 4.79, N, 24.99. **Found:** C, 60.52, H, 4.83, N, 24.55%.^1^H NMR (DMSO-d_6_); δ 3.60 (br, 1H, NH), 6.20 (s, 1H, C4), 6.70-7.30 (dd, 4H, Ar), 7.70 (s, 1H, -N=CH), 9.70 (br, 1H, OH), 11.30 (br, 1H, NH).^13^C NMR (100 MHz DMSO-d_6_): δ 77.90, 115.32, 127.16, 130.08, 139.48, 157.18, 160.36. IR (KBr): υ(cm^1−^) 3324, 1604, 1455, 1259.

**2h: Solid 92% mp 64–67°C. Anal. Calc. for C_17_H_16_N_6_:** C, 67.09, H, 5.30, N, 27.61. **Found:** C, 66.90, H, 5.41, N, 27.34%.^1^H NMR (DMSO-d_6_); δ 4.30 (br, 1H, NH), 6.30 (s, 1H, C4), 7.40-7.90 (m, 5H, Ar), 8.75 (s, 1H, -N=CH), 11.30 (br, 1H, NH). ^13^C NMR (100 MHz DMSO-d_6_): δ 77.44, 125.02, 128.87, 131.32, 133.76, 161.46. IR (KBr): υ(cm^1−^) 3166, 3050, 1619, 1483, 1304.

### Synthesis of 3, 5-bis(substituted) isoxazoles (3a-c) - General procedure

A mixture of (1a, 0.01mol) and an equimoler amount of hydroxylamine hydrochloride were taken in autoclave reaction container (Teflon liner). The lid was placed and was lowered into the autoclave, the plates were kept over it and the autoclave was closed and tightened. It was kept in the oven at150°C for 3hour. The products after cooling, were extracted into ethylacetate (20 ml), washed thoroughly with water. The organic layer was dried over Na_2_SO_4_, solvent was evaporated and the product obtained as:

**3a: Solid 80% mp 112–113°C. Anal. Calc. for C_17_H_13_C_l2_N_5_O:** C, 64.43, H, 6.44, N, 25.05. **Found:** C, 64.21, H, 6.53, N, 24.91%.^1^H NMR (CDCl_3_); δ 3.00 (s, 6H, -N(CH_3_)_2_), 6.70-7.70 (dd, 4H, Ar), 8.60 (s, 1H, -N=CH).^13^C NMR (100 MHz CDCl_3_): δ 40.19, 98.60 111.73, 122.24, 129.87, 147.43, 150.23, 152.10, 160.82. IR (KBr): υ(cm^1−^) 3254, 2921, 2817, 1612, 1531, 1450.

**3b: Solid 75% mp 78–80°C. Anal. Calc. for C_23_H_27_N_5_O_7_:** C, 56.90, H, 5.61, N, 14.43. **Found:** C, 56.85, H, 5.75, N, 14.15%.^1^H NMR (DMSO-d_6_); δ 3.80 (s, 9H, -OCH_3_), 6.70 (s, 1H, C4), 6.90 (s, 2H, Ar), 8.00 (s, 1H, -N=CH).^13^C NMR (100 MHz DMSO-d_6_): δ 55.90, 60.06, 103.58, 128.56, 138.53, 144.42, 147.84, 152.38, and 153.49. IR (KBr): υ(cm^1−^) 3571, 3286, 2952, 1594, 1474.

**3c: Solid 77% mp 92–94°C. Anal. Calc. for C_17_H_13_C_l2_N_5_O:** C, 54.56, H, 3.50, N, 18.71. **Found:** C, 54.43, H, 3.61, N, 18.60%.^1^H NMR (CDCl_3_); δ 4.10 (br, 1H, NH), 6.80 (s, 1H, C4), 7.90-8.20 (dd, 4H, Ar), 8.70 (s, 1H, -N=CH), 8.90 (s, 1H, -N=CH). ^13^C NMR (100 MHz CDCl_3_): δ 98.59, 123.96, 127.30, 131.46, 146.79, 147.47, 155.50. IR (KBr): υ(cm^1−^) 3318, 3108, 2947, 1601, 1335.
